# The Proposition Stated

**Published:** 1912-08

**Authors:** 


### The Proposition Stated.

Today society is face to face with the fact that in spite of our marvelous advancement along commercial and social lines and the tendency towards higher moral standards, the race is surely and steadily degenerating. If this be true, the question then arises: can society avert this impending disaster? Or, must it follow the path of all preceding civilizations, save that of the Jewish race?

Before this question is answered, let it be understood that society today differs from that existing at any other period of the world's history in this particular function, that it possesses the knowledge by which degeneracy can be stamped out and a nobler and a better race propagated at will.

This being true, the character of future generations rests upon the attitude and actions of the present. Then, assuming this responsibility, the question resolves itself into one of expediency; whether action should be compelled by social reform, or by law?

